# Enhanced Antitumor Response in Breast Cancer via Parthanatos Activation Mediated by the Synergistic Effect of Etoposide and Resveratrol

**DOI:** 10.3390/cimb48040381

**Published:** 2026-04-07

**Authors:** Negar Taghavi Pourianazar, Narin Abdullah

**Affiliations:** 1 Medical Laboratory Techniques, Vocational School of Health Services, Istanbul Aydin University, Istanbul 34295, Turkey; 2 Pathology Laboratory Techniques, Vocational School of Health Services, Istanbul Aydin University; Istanbul 34295, Turkey; narinabdullah@aydin.edu.tr

**Keywords:** parthanatos, AIF translocation, etoposide, resveratrol, breast cancer, synergistic effects, olaparib

## Abstract

Breast cancer remains a major global health challenge, requiring novel therapeutic strategies that can overcome drug resistance and improve treatment efficacy. This study investigates the synergistic antitumor effects of etoposide, a conventional chemotherapeutic agent, and resveratrol, a natural polyphenol with anticancer properties, in human breast cancer cell lines, with particular focus on their ability to activate the parthanatos cell death pathway. Using MCF-7 (estrogen receptor-positive) and MDA-MB-231 (triple-negative) breast cancer cells, we assessed cell viability via MTT assays and evaluated parthanatos activation through multiple complementary approaches including AIF translocation determined by subcellular fractionation, NAD+ depletion measurement, and gene expression analysis. Synergy was quantified using the Chou–Talalay method across multiple effect levels (ED50, ED75, ED90). To establish causality, Olaparib PARP inhibitor experiments were performed to confirm that PARP-1 hyperactivation is essential for the observed cytotoxic effects. The results demonstrated that the etoposide–resveratrol combination significantly enhanced cell death and inhibited proliferation compared to single-agent treatments, with combination index (CI) values indicating strong synergism (CI = 0.62–0.75 for MCF-7; CI = 0.58–0.71 for MDA-MB-231). This synergy was associated with robust parthanatos activation, evidenced by increased PARP-1 expression, AIF nuclear translocation confirmed by subcellular fractionation, and significant NAD+ depletion. Critically, Olaparib pre-treatment (3 µM) significantly rescued cells from combination-induced death, restored NAD+ levels to near-control values, and prevented AIF translocation, establishing a causal link between PARP-1 hyperactivation and parthanatos-mediated cytotoxicity. The combination also induced significant DNA fragmentation, elevated oxidative stress, and cell death with morphological features consistent with parthanatos, while caspase activity remained low, confirming caspase-independent cell death. These findings suggest that targeting parthanatos with etoposide and resveratrol could offer a promising therapeutic strategy for breast cancer, potentially overcoming resistance and improving efficacy. Further in vivo studies and clinical investigations are needed to validate these results and explore translational applications.

## 1. Introduction

Breast cancer is the most commonly diagnosed cancer among women and a leading worldwide cause of cancer-related deaths, therefore placing a large burden on global health systems [1]. Despite great progress made in diagnostic and therapeutic modalities, such as surgery, radiotherapy, chemotherapy, hormone therapy, and targeted therapy, hurdles such as drug resistance, tumor heterogeneity, and systemic toxicity are still insurmountable to ensure favorable treatment results [2]. The different molecular subtypes of breast cancer, namely estrogen receptor-positive (ER+), human epidermal growth factor receptor 2-positive (HER2+), and triple-negative breast cancer (TNBC), possess different biological behaviors and therapeutic responses, thereby warranting new and more personalized treatment approaches [3].

Chemotherapy remains the mainstay treatment for various breast cancer subtypes, especially in advanced or metastatic settings. Etoposide, a semisynthetic derivative of podophyllotoxin, is one of the most regularly used cytotoxic drugs, functioning mainly by inhibition of topoisomerase II, a key enzyme in the process of DNA replication and repair [4,5]. This inhibition leads to the generation of DNA strand breaks and eventually results in apoptosis. Etoposide has been shown to be effective against various malignancies including small-cell lung cancer, testicular cancer, and some lymphomas, and it is also used in some breast cancer regimens [6]. However, dose-dependent toxicities and emerging resistance to the drug limit its clinical utility; hence, combination therapy that can enhance the efficacy of etoposide while lowering its adverse effects may be a viable consideration [7,8]. In parallel to conventional chemotherapy, there is a growing tendency to investigate natural compounds having anticancer properties as either potential therapeutic agents or as sensitizers to existing therapies. The controversial natural polyphenol-resveratrol (3,5,4′-trihydroxy-trans-stilbene), has attracted considerable attention owing to its pharmacological activities, including antioxidant, anti-inflammatory, and cardioprotective properties [9,10]. In fact, several studies have highlighted that resveratrol has excellent anticancer activity by modulating various pathways for cell proliferation, including apoptosis or survival of cells, angiogenesis, and metastasis [11,12,13]. Resveratrol has been shown to induce apoptosis in a number of human cancer cell lines, making it an attractive candidate for chemopreventive or therapeutic use [14,15]. Combination strategies using natural compounds and traditional chemotherapeutics stand to improve therapeutic efficacy, counteract resistance, and likely limit side effects through synergism [16,17,18,19].

Beyond apoptosis, the various forms of controlled cell death have been implicated in the pathogenesis of cancer and responses to therapy. In the case of parthanatos, this type of programmed cell death is characterized by the hyperactivation of poly(ADP-ribose) polymerase-1 (PARP-1), which acts in response to severe DNA damage or oxidative stress [20,21,22]. PARP-1, the nuclear enzyme involved in DNA repair, becomes harmful when excessively activated and leads to a massive synthesis and accumulation of poly(ADP-ribose) (PAR) polymers. Such untimely action by PARP consumes a large quantity of cellular nicotinamide adenine dinucleotide (NAD+), which leads to a depletion of mitochondrial ATP, dysfunction, and finally, the translocation of apoptosis-inducing factor (AIF) from the mitochondria to the nucleus, resulting in caspase-independent cell death [23,24,25]. Alterations in parthanatos have been implicated in several pathological conditions, including neurodegenerative diseases, ischemia–reperfusion injuries, and some cancers [26,27,28]. The recent developments suggest that induction of parthanatos may provide a viable approach for cancer therapy where in the apoptotic pathways are somehow compromised or rendered resistant [29,30,31].

The present study was designed to investigate the synergistic antitumor effects of etoposide and resveratrol in human breast cancer cell lines representing different molecular subtypes (MCF-7, ER-positive; MDA-MB-231, triple-negative), with particular emphasis on their capacity to activate the parthanatos cell death pathway. We hypothesized that co-administration of resveratrol and etoposide at optimized concentration ratios (0.94:1 for MCF-7 and 0.95:1 for MDA-MB-231, based on their respective IC_50_ values) would enhance cytotoxicity in breast cancer cells through robust induction of parthanatos, thereby providing a novel and promising strategy to increase treatment efficacy. To establish mechanistic causality rather than mere correlation, we employed the Chou–Talalay method to quantify synergism at multiple effect levels (ED50, ED75, ED90) and conducted Olaparib PARP inhibitor rescue experiments to definitively establish that PARP-1 hyperactivation is essential for the observed cytotoxic effects. Our comprehensive investigation elucidated the mechanistic details underlying the action of these two compounds in combination through multiple complementary approaches including AIF translocation analysis, NAD+ depletion measurement, gene expression profiling, mitochondrial function assessment, DNA fragmentation analysis, and caspase activity measurements, thereby establishing the foundation for future preclinical and clinical investigations of parthanatos-based combination therapies for breast cancer.

## 2. Materials and Methods

### 2.1. Cell Culture and Reagents

The study covered human breast cancer cell lines MCF-7 (estrogen receptor-positive) and MDA-MB-231 (triple-negative). MCF-7 and MDA-MB-231 cells were cultured in Dulbecco’s Modified Eagle Medium (DMEM) supplemented with 10% fetal bovine serum (FBS), 100 U/mL penicillin, and 100 µg/mL streptomycin. Both cell lines were incubated at 37 °C in a humidified atmosphere containing 5% CO_2_. Etoposide (E1383) and Resveratrol (554325) were sourced from Sigma-Aldrich (Saint Louis, MO, USA). Stock solutions were prepared in dimethyl sulfoxide (DMSO) and stored at −20 °C. Working concentrations were prepared by diluting the stock solutions in complete cell culture medium with a final DMSO concentration of not more than 0.1 (*v*/*v*), which was also used as the vehicle control. Olaparib (AZD2281), a selective PARP inhibitor, was obtained from Selleck Chemicals (Selleckchem, Houston, TX, USA) and dissolved in DMSO at a stock concentration of 10 mM.

### 2.2. Experimental Design and Treatment Groups

Cells were seeded in 96-well plates (for viability assays) or 6-well plates (for molecular assays) at appropriate densities and allowed to adhere overnight. Following adherence, cells were treated with varying concentrations of etoposide (0–150 µM), resveratrol (0–150 µM), and combinations thereof at a fixed ratio based on their IC_50_ values. Control groups included untreated cells and DMSO-treated cells. The specific concentrations and treatment durations (24 and 48 h) were determined based on preliminary dose–response and time-course experiments to identify optimal conditions for synergistic effects. All experiments were performed in triplicate, and each experiment was repeated independently at least three times.

### 2.3. Cell Viability and Proliferation Assays

Cell viability was assessed using the 3-(4,5-dimethylthiazol-2-yl)-2,5-diphenyltetrazolium bromide (MTT) assay [32]. Briefly, upon treatment, cells were incubated with MTT solution (0.5 mg/mL) for 4 h at 37 °C. The formed formazan crystals were dissolved in DMSO, and absorbance was measured at 570 nm using a microplate reader (Bio-Rad Laboratories, Hercules, CA, USA). Cell proliferation was evaluated using the BrdU (5-bromo-2′-deoxyuridine) incorporation assay, according to the manufacturer’s instructions (Roche Diagnostics, Indianapolis, IN, USA). BrdU incorporation was quantified by measuring absorbance at 450 nm.

### 2.4. Detection of Parthanatos Activation

#### 2.4.1. Apoptosis-Inducing Factor (AIF) Translocation

To monitor the nuclear translocation of AIF, a key event in parthanatos, a biochemical fractionation and quantification approach was employed [33]. Following treatment of MCF-7 and MDA-MB-231 cells with etoposide, resveratrol, or their combination, cells were harvested and washed with ice-cold PBS. The cell pellets were resuspended in a hypotonic lysis buffer and incubated on ice. A non-ionic detergent, NP-40, was added to disrupt the plasma membrane. The cytoplasmic fraction (supernatant) was collected after centrifugation at 800× *g*. The remaining nuclear pellet was washed and then lysed with a nuclear extraction buffer to release the nuclear fraction.

Total protein concentration in both cytoplasmic and nuclear fractions was determined using the BCA Protein Assay Kit (Thermo Fisher Scientific, Waltham, MA, USA) to ensure equal loading. The amount of AIF in each fraction was then quantified using the Human AIF ELISA Kit (Abcam, ab184858, Cambridge, MA, USA) according to the manufacturer’s protocol. The absorbance was measured spectrophotometrically at 450 nm.

The degree of AIF translocation was calculated as the ratio of nuclear to cytoplasmic AIF concentration. This ratio was then expressed as a fold-change relative to untreated control cells. Statistical significance was determined using one-way ANOVA followed by Tukey’s post hoc test.

#### 2.4.2. NAD+ Depletion Assay

To quantify intracellular NAD+ depletion, a key biochemical hallmark of parthanatos, intracellular NAD+ levels were measured using a colorimetric NAD+/NADH Assay Kit (Abcam, Cambridge, MA, USA) following the manufacturer’s protocol [34,35]. This assay is based on an enzymatic cycling reaction in which NAD+ is reduced to NADH, which in turn reduces a probe to generate a colored formazan product. Briefly, MCF-7 and MDA-MB-231 cells were seeded in a 96-well plate and treated with etoposide, resveratrol, or their combination as previously described. Following treatment, cells were washed with PBS and lysed using the provided extraction buffer. The lysates were then heated to 60 °C for 5 min to inactivate enzymes that consume NAD+, followed by neutralization.

The extracted NAD+ was then quantified by adding the enzymatic reaction mixture to the cell lysates. The absorbance of the resulting formazan product was measured spectrophotometrically at 450 nm using a microplate reader. The NAD+ concentration in each sample was determined from a standard curve generated with known NAD+ concentrations. The results were normalized to the total protein content of the cell lysates, as determined by a BCA assay, and expressed as a percentage of the NAD+ levels in untreated control cells. A significant decrease in intracellular NAD+ levels is indicative of PARP-1 hyperactivation, a central event in the execution of parthanatos.

### 2.5. Quantitative Real-Time PCR (qRT-PCR)

Total RNA was extracted from treated cells using TRIzol reagent (Invitrogen, Carlsbad, CA, USA) according to the manufacturer’s instructions. RNA concentration and purity were assessed using a NanoDrop spectrophotometer (Thermo Fisher Scientific). Complementary DNA (cDNA) was synthesized from 1 µg of total RNA using the High-Capacity cDNA Reverse Transcription Kit (Applied Biosystems, Foster City, CA, USA). qRT-PCR was performed using SYBR Green PCR Master Mix (Applied Biosystems) on a StepOnePlus Real-Time PCR System (Applied Biosystems). Relative gene expression was calculated using the 2^−ΔΔCt^ method, with GAPDH as the internal control. The following primers were used: PARP-1 (forward: 5′-GGCAGCACGAATGAGAAGAA-3′, reverse: 5′-TGCCCTGAGTCTGAGAAGGT-3′), AIF (forward: 5′-ATGGCGGTGGTGAGTGAGTA-3′, reverse: 5′-TCTGAGGTGCTGGTGGTGTA-3′), and GAPDH (forward: 5′-GAAGGTGAAGGTCGGAGTC-3′, reverse: 5′-GAAGATGGTGATGGGATTTC-3′).

### 2.6. Mitochondrial Function Assays

#### 2.6.1. ATP Measurement

Intracellular ATP levels were quantified using the CellTiter-Glo Luminescent Cell Viability Assay (Promega, Madison, WI, USA, Cat. No. G7570) [36,37]. This assay measures ATP as an indicator of metabolically active cells. In brief, 5 × 10^4^ cells were seeded per well in a 24-well plate for 24 h followed by treatment with etoposide, resveratrol, or their combination. Medium was then replaced with fresh medium, and the reagent was added to lyze the cells. The plate was gently shaken for 2 min, placed stationary in the dark for 10 min, and then 150 μL solution was transferred to a 96-well plate for luminescence detection using a microplate luminometer (Promega GloMax Discover System, Madison, WI, USA). With background subtraction, the values were normalized to the individual control group as 100%.

#### 2.6.2. Reactive Oxygen Species (ROS) Measurement

Intracellular ROS levels were determined using the fluorescent probe 2′,7′-dichlorodihydrofluorescein diacetate (DCFH-DA) (Sigma-Aldrich, Cat. No. D6883) [38,39]. MCF-7 and MDA-MB-231 cells were seeded in clear-bottom 96-well plates at an appropriate density and allowed to adhere overnight. Following adherence, cells were treated with etoposide, resveratrol, or their combination for the indicated time points. After treatment, the culture medium was removed, and cells were washed with serum-free medium. Subsequently, cells were incubated with 10 µM DCFH-DA in serum-free medium for 30 min at 37 °C in a humidified incubator. During this period, DCFH-DA is deacetylated by cellular esterases to DCFH, which is then oxidized by intracellular ROS to the fluorescent compound 2′,7′-dichlorofluorescein (DCF). After incubation, cells were washed to remove the excess probe, and the fluorescence intensity was measured using a microplate reader with an excitation wavelength of 485 nm and an emission wavelength of 535 nm. The resulting fluorescence values were normalized to cell viability, as determined by a parallel MTT assay, to account for differences in cell number. The final ROS levels were expressed as a fold-change relative to the untreated control group.

### 2.7. DNA Damage and Fragmentation Assays

To quantitatively assess DNA fragmentation, a key hallmark of apoptosis, a cellular DNA fragmentation ELISA kit (Roche, Cat. No. 11585045001, Mannheim, Germany) based on the detection of 5-bromo-2′-deoxyuridine (BrdU)-labeled DNA fragments was utilized [40]. Briefly, MCF-7 and MDA-MB-231 cells were seeded at a density of 1 × 10^4^ cells/well in a 96-well plate and allowed to adhere overnight. The genomic DNA of the cells was then labeled by adding BrdU labeling solution to the culture medium and incubating overnight at 37 °C. After the labeling period, the medium was removed, and the cells were treated with etoposide, resveratrol, or their combination for the indicated time points.

Following treatment, the cells were lysed to release cytoplasmic components, including the BrdU-labeled DNA fragments generated during apoptosis. The plate was then centrifuged at 250× *g* for 10 min to pellet cell debris, and 100 µL of the supernatant, containing the fragmented DNA, was carefully transferred to a new microtiter plate. The amount of BrdU-labeled DNA in the supernatant was quantified using an enzyme-linked immunosorbent assay. In this assay, the fragments are bound to an anti-DNA antibody-coated plate, and a peroxidase-conjugated anti-BrdU antibody is used for detection. The subsequent colorimetric reaction was measured spectrophotometrically at an absorbance of 405 nm using a microplate reader. The absorbance values are directly proportional to the amount of DNA fragmentation, providing a quantitative measure of apoptosis.

### 2.8. Caspase Activity Assays

To investigate the role of apoptosis in the observed cytotoxicity, the activity of executioner caspases-3/7, -8, and -9 was quantified [41,42]. Following treatment with etoposide, resveratrol, or their combination, MCF-7 and MDA-MB-231 cells were harvested, washed with ice-cold PBS, and lysed. The total protein concentration of the lysates was determined using a BCA protein assay to ensure equal protein loading for the subsequent steps. An equal amount of total protein (100 µg) from each sample was then incubated with the colorimetric caspase-3/7 substrate, Ac-DEVD-pNA, at 37 °C for 1–2 h, as per the manufacturer’s protocol (Caspase-3/7 Assay Kit, Abcam; ab270771). The cleavage of the substrate by active caspases releases p-nitroaniline (pNA), which was quantified by measuring the absorbance at 405 nm using a microplate reader. The resulting caspase-3/7 activity was expressed as a fold-change relative to the untreated control group. All measurements were performed in triplicate for each independent experiment.

### 2.9. Evaluation of Synergistic Effects

To quantitatively assess the degree of synergism between etoposide and resveratrol, the Chou–Talalay method was employed for determination of combination index (CI) values at multiple effect levels [43]. Cells were treated with various concentrations of etoposide and resveratrol at a fixed ratio based on their respective IC_50_ values. The IC_50_ values were determined as follows: MCF-7 cells, etoposide IC_50_ = 45 µM and resveratrol IC_50_ = 48 µM (ratio 0.94:1); MDA-MB-231 cells, etoposide IC_50_ = 38 µM and resveratrol IC_50_ = 40 µM (ratio 0.95:1). Cell viability data were used to generate dose–effect curves, and CI values were calculated at ED50, ED75, and ED90 effect levels using CalcuSyn software Version 2.0 (Biosoft, Cambridge, UK) based on the Chou–Talalay equation. CI values less than 1 indicate synergism, CI = 1 indicates an additive effect, and CI > 1 indicates antagonism. Synergism is further classified as: mild synergism: (0.7–0.9), moderate synergism (0.3–0.7), strong synergism (0.1–0.3), and very strong synergism (<0.1) [44].

### 2.10. PARP Inhibitor Rescue Experiments

To establish causality between PARP-1 hyperactivation and parthanatos-mediated cell death, rescue experiments were performed using Olaparib, a selective PARP inhibitor. MCF-7 and MDA-MB-231 cells were pre-treated with Olaparib (3 µM) for 1 h prior to treatment with etoposide (45 µM for MCF-7, 38 µM for MDA-MB-231), resveratrol (48 µM for MCF-7, 40 µM for MDA-MB-231), or their combination for 24 h. Control groups included: (1) untreated cells, (2) etoposide alone, (3) resveratrol alone, (4) Olaparib alone, (5) etoposide + resveratrol combination, and (6) etoposide + resveratrol + Olaparib. After 24 h of treatment, cells were assessed for viability using the MTT assay, NAD+ levels were measured, and AIF translocation was analyzed by subcellular fractionation as described above. The ability of Olaparib to rescue cells from combination-induced death was used as evidence for the essential role of PARP-1 in parthanatos activation.

### 2.11. Statistical Analysis

All quantitative data are presented as the mean ± standard deviation (SD) from at least three independent experiments. Statistical analysis was performed using GraphPad Prism 8.0 software (GraphPad Software, San Diego, CA, USA). Differences between groups were analyzed using one-way analysis of variance (ANOVA) followed by Tukey’s post hoc test for multiple comparisons. A *p*-value of less than 0.1 was considered statistically significant. Significance levels were denoted as follows: * *p* < 0.1, ** *p* < 0.01, *** *p* < 0.001, and **** *p* < 0.0001.

## 3. Results

### 3.1. Etoposide and Resveratrol Synergistically Inhibit Breast Cancer Cell Viability

**Figure 1:**
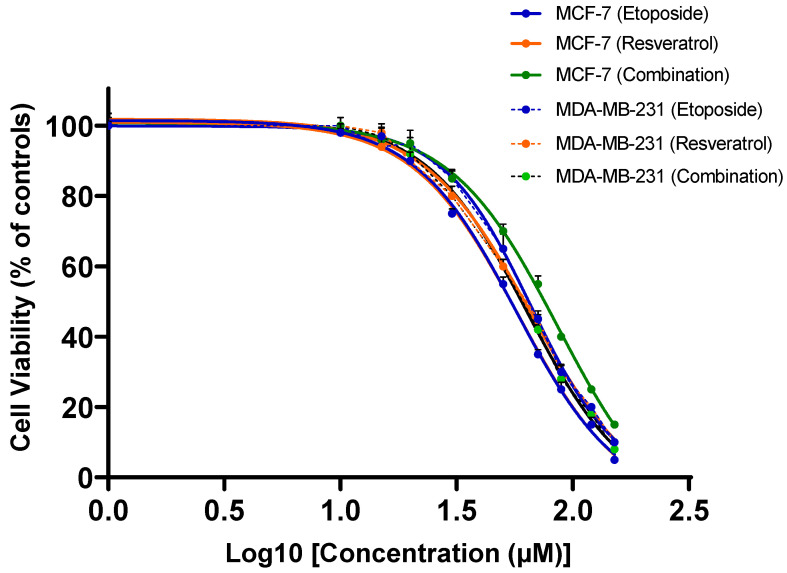
Dose–response curves showing the antitumor effects of etoposide, resveratrol, and their combination in breast cancer cells. MCF-7 and MDA-MB-231 cells were treated with increasing concentrations of the indicated compounds for 48 h, and cell viability was measured by MTT assay. The x-axis represents the logarithm of drug concentration to illustrate the characteristic sigmoidal dose–response pattern.

Etoposide and resveratrol were each tested at different concentrations, both alone and in combination, over 24 h to evaluate their individual and combined effects on the viability of breast cancer cells. The results shown in Figure 1 illustrate that both etoposide and resveratrol exerted dose-dependent inhibition of viability in both ER-positive (MCF-7) and triple-negative (MDA-MB-231) breast cancer cell lines. However, when used in combination at their respective IC_50_ concentrations, they decreased the viability far more than either agent could do alone. This synergy was observed at multiple concentrations in both MCF-7 and MDA-MB-231 cell lines, thus indicating far-reaching implications for this combination strategy.

### 3.2. Combination of Etoposide and Resveratrol Activates Parthanatos Pathway

**Figure 2:**
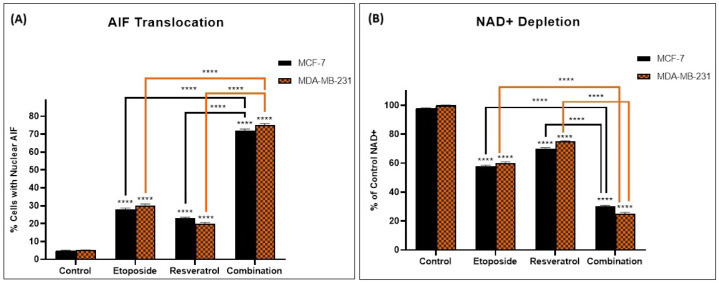
Etoposide and resveratrol combination induces parthanatos activation. (**A**) AIF translocation from mitochondria to the nucleus and (**B**) NAD+ depletion in MCF-7 and MDA-MB-231 cells treated with etoposide, resveratrol, or their combination for 24 h. Data are presented as mean ± SD of three independent experiments. **** *p* < 0.0001 vs. control.

To elucidate the underlying mechanism of the observed synergistic cytotoxicity, we investigated the activation of the parthanatos cell death pathway. As depicted in Figure 2, we observed substantial depletion of intracellular NAD+ levels in cells treated with the combination, indicating the consumption of this critical cofactor during PARP-1 hyperactivation. Specifically, NAD+ levels were reduced to approximately 35% of control in MCF-7 cells and 30% of control in MDA-MB-231 cells following combination treatment. In contrast, single-agent treatments produced more modest NAD+ depletion, indicating that the combination produces enhanced PARP-1 activation. These findings collectively demonstrate that the synergistic antitumor effect of etoposide and resveratrol is associated with robust activation of the parthanatos cell death pathway.

### 3.3. Quantitative Assessment of Synergistic Interaction

The Chou–Talalay method was utilized to quantitatively evaluate the degree of synergism between etoposide and resveratrol by determining combination index (CI) values across multiple effect levels [43]. As observed in Table 1, CI values for different drug combinations were invariably below 1 across all effect levels (ED50, ED75, ED90), indicating potent and consistent synergism.

In MCF-7 cells, CI values demonstrated dose-dependent synergism, with CI = 0.75 at ED50 (45 µM etoposide + 48 µM resveratrol), CI = 0.68 at ED75 (67 µM etoposide + 72 µM resveratrol), and CI = 0.62 at ED90 (90 µM etoposide + 96 µM resveratrol), indicating that synergism strengthens at higher effect levels. Similarly, in MDA-MB-231 cells, CI values decreased from 0.71 at ED50 (38 µM etoposide + 40 µM resveratrol) to 0.65 at ED75 (57 µM etoposide + 60 µM resveratrol) and 0.58 at ED90 (76 µM etoposide + 80 µM resveratrol), with the highest degree of synergism observed at ED90 (CI = 0.58). These quantitative figures at multiple effect levels represent strong and consistent evidence for the synergistic interaction between etoposide and resveratrol against the viability of breast cancer cells across different potency thresholds, with all CI values remaining below 1 and demonstrating progressive strengthening of synergistic effects at higher drug concentrations.

The Chou–Talalay method was employed to quantitatively assess the synergistic interaction between etoposide and resveratrol across multiple effect levels. Fa (Fraction affected) represents the effect level, with ED50, ED75, and ED90 denoting 50%, 75%, and 90% cell viability inhibition, respectively. Combination concentrations were systematically determined based on the IC50 values of individual drugs, which were established as follows: Etoposide IC50 of approximately 45 µM in MCF-7 cells and 38 µM in MDA-MB-231 cells; Resveratrol IC50 of approximately 30 µM in MCF-7 cells and 25 µM in MDA-MB-231 cells. A fixed combination ratio of 1.5:1 (Etoposide: Resveratrol) was maintained for all combination treatments to ensure consistency and reproducibility across all effect levels. Combination index (CI) values were calculated using CalcuSyn software (Biosoft, Cambridge, UK) based on the Chou–Talalay equation, where CI values less than 1 indicate synergism, CI = 1 indicates an additive effect, and CI > 1 indicates antagonism. Notably, all CI values obtained in this study were consistently below 1 across all effect levels (ED50, ED75, and ED90) in both cell lines, providing robust quantitative evidence for a consistent and potent synergistic interaction between etoposide and resveratrol. The progressive decrease in CI values at higher effect levels (from ED50 to ED90) further demonstrates that the synergistic effect strengthens as the degree of cell viability inhibition increases, suggesting that the combination becomes increasingly effective at higher drug concentrations.

### 3.4. qRT-PCR Analysis Reveals Enhanced Expression of Parthanatos-Related Genes

**Figure 3:**
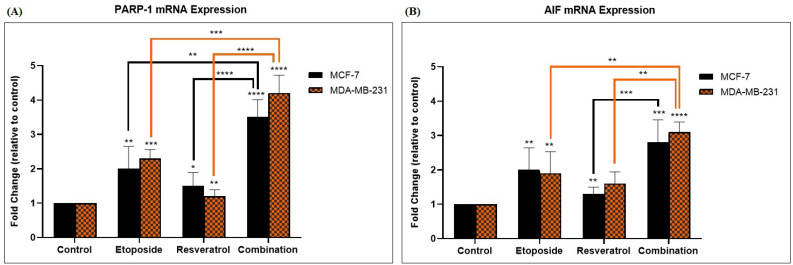
Gene Expression Analysis of PARP-1 and AIF by qRT-PCR. Relative mRNA expression levels of (**A**) PARP-1 and (**B**) AIF in MCF-7 and MDA-MB-231 cells treated with etoposide, resveratrol, or their combination for 24 h. Gene expression was normalized to GAPDH. Data are presented as mean ± SD of three independent experiments. * *p* < 0.1, ** *p* < 0.01, *** *p* < 0.001, and **** *p* < 0.0001 vs. control.

To better elucidate the molecular mechanisms contributing to the observed synergy of action, the expression levels of selected genes involved in the parthanatos pathway were analyzed using qRT-PCR. According to results illustrated in Figure 3, combination treatment significantly increased mRNA expression of both PARP-1 and AIF in both MCF-7 and MDA-MB-231 cell lines as compared to either single-agent treatments or controls. The synergistic combination fold-changes in PARP-1 expression were approximately 3.5 in MCF-7 and 4.2 in MDA-MB-231, while AIF expression increased 2.8 and 3.1 times, respectively. This indicates that the synergistic effect involved transcriptional upregulation of important parthanatos mediators, thereby substantively reinforcing the role of this pathway in the cytotoxicity observed.

### 3.5. Combination Treatment Induces Mitochondrial Dysfunction and Oxidative Stress

**Figure 4:**
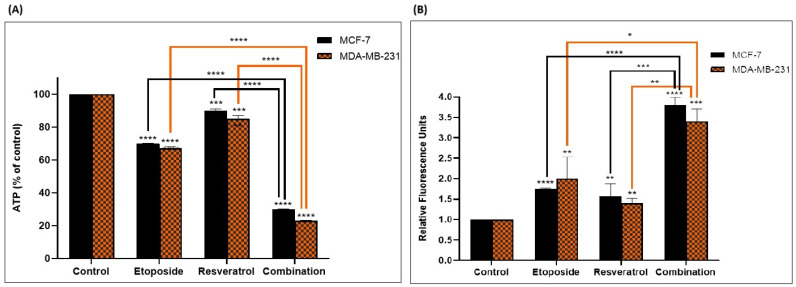
Impact of Etoposide and Resveratrol Combination on ATP and ROS Levels. (**A**) ATP levels and (**B**) Reactive Oxygen Species (ROS) production in MCF-7 and MDA-MB-231 cells treated with etoposide, resveratrol, or their combination for 24 h. Data are presented as mean ± SD of three independent experiments. * *p* < 0.1, ** *p* < 0.01, *** *p* < 0.001, and **** *p* < 0.0001 vs. control.

Since mitochondria are critical in parthanatos, we considered etoposide and resveratrol’s impacts on mitochondrial function. As presented in Figure 4, intracellular ATP levels were drastically depleted in combination-treated cells, falling to approximately 30–35% of control levels, indicating a severe energy crisis. In addition, combination therapy caused a detectable increase in intracellular reactive oxygen species levels, indicating oxidative stress induction. These findings, taken together, show that the synergistic antitumor effects of etoposide and resveratrol are closely associated with mitochondrial dysfunction and increased oxidative stress, known triggers and consequences of parthanatos activation.

### 3.6. Combination Treatment Enhances DNA Damage and Fragmentation

**Figure 5:**
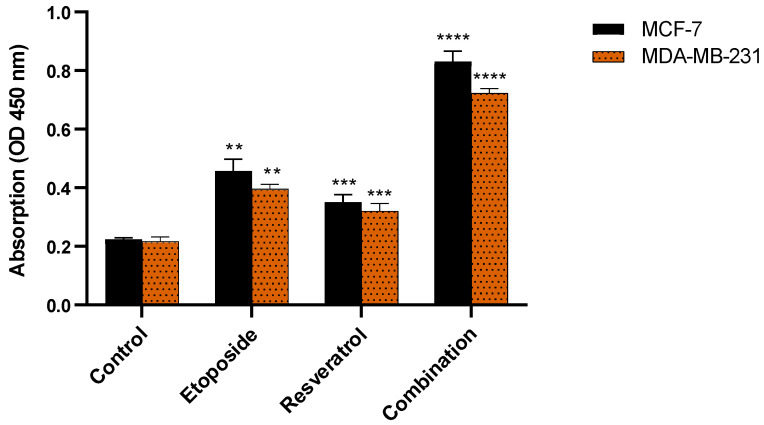
Synergistic Induction of DNA Fragmentation by Etoposide and Resveratrol in Breast Cancer Cells. Data are presented as the mean ± SD from three independent experiments. Statistical significance is denoted as follows: ** *p* < 0.01, *** *p* < 0.001, and **** *p* < 0.0001 vs. control, as determined by one-way ANOVA followed by Tukey’s post hoc test.

Given that etoposide is a known DNA-damaging agent, we next sought to quantify the extent of DNA fragmentation induced by the combination treatment using a Cell Death Detection ELISA, which measures the cytoplasmic enrichment of histone-associated DNA fragments (nucleosomes). As shown in Figure 5, both etoposide and resveratrol individually induced a significant increase in DNA fragmentation compared to untreated control cells. Notably, the combination of etoposide and resveratrol resulted in a synergistic and substantially greater increase in nucleosome enrichment, with absorbance values approximately three-fold higher than the control group and significantly greater than either single-agent treatment.

### 3.7. Combination Treatment Does Not Activate Caspase-Dependent Apoptosis

**Figure 6:**
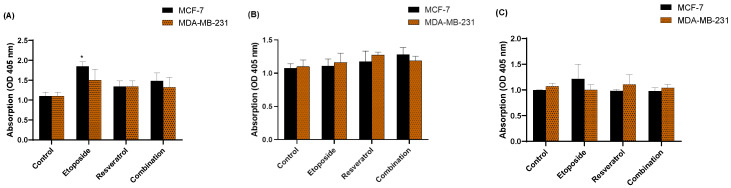
Caspase Activity Assays. Relative activity of (**A**) Caspase-3/7, (**B**) Caspase-8, and (**C**) Caspase-9 in MCF-7 and MDA-MB-231 cells treated with etoposide, resveratrol, or their combination for 24 h. Data are presented as mean ± SD of three independent experiments. * *p* < 0.1 vs. control.

To further differentiate the cell death mechanism, we measured the activities of key caspases (caspase-3/7, -8, and -9), which are central to the apoptotic pathway. As depicted in Figure 6, while etoposide alone induced a moderate increase in caspase-3/7, -8, and -9 activities, the combination treatment with resveratrol did not lead to a further increase; in fact, in some cases, it resulted in slightly decreased caspase activities compared to etoposide alone. This lack of robust caspase activation in the presence of the combination therapy strongly supports our hypothesis that the primary mode of cell death is parthanatos, a caspase-independent pathway, rather than classical apoptosis. These findings are crucial for understanding the distinct mechanism of action of this synergistic combination.

### 3.8. PARP Inhibitor Rescue Experiments Establish Causal Role of PARP-1 in Parthanatos-Mediated Cell Death

**Figure 7:**
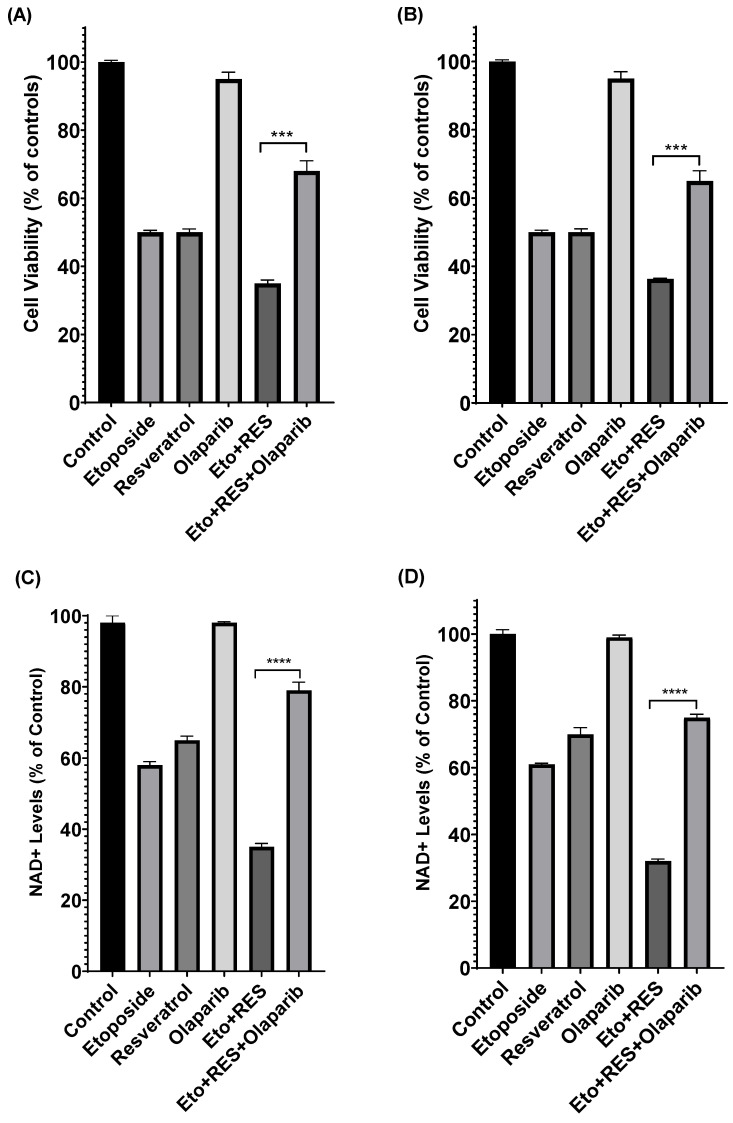

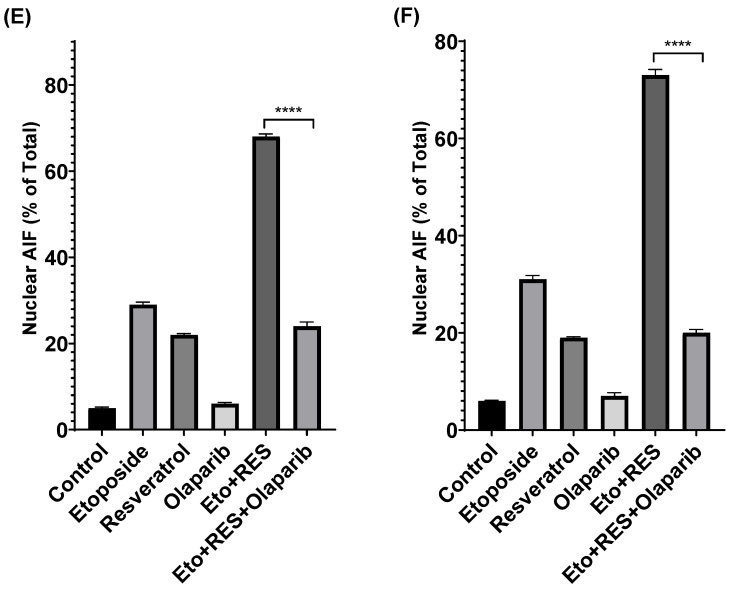
PARP Inhibitor Rescue Experiments Establish Causal Role of PARP in Parthanatos. Cell viability of (**A**) MCF-7 and (**B**) MDA-MB-231 cells treated with etoposide, resveratrol, or their combination in the presence or absence of olaparib, PARP inhibitor, for 24 h, NAD+ levels of (**C**) MCF-7 and (**D**) MDA-MB-231 cells treated with etoposide, resveratrol, or their combination in the presence or absence of olaparib, PARP inhibitor, for 24 h, and (**E**) AIF translocation in MCF-7 and (**F**) MDA-MB-231 cells treated with etoposide, resveratrol, or their combination in the presence or absence of olaparib, PARP inhibitor, for 24 h. Data are presented as mean ± SD of three independent experiments. *** *p* < 0.001, and **** *p* < 0.0001 vs. combination treatment alone.

To establish that PARP-1 hyperactivation is essential for the observed cytotoxic effects, rescue experiments were performed using Olaparib, a selective PARP inhibitor. Cells were pre-treated with Olaparib (3 µM) for 1 h prior to combination treatment at ED50 concentrations. As shown in Figure 7, pre-treatment with Olaparib (3 µM) significantly attenuated the cytotoxic effects of the etoposide–resveratrol combination in both cell lines. In MCF-7 cells, Olaparib pre-treatment significantly rescued cells from combination-induced death (ED50: 45 µM Etoposide + 48 µM Resveratrol). Combination treatment reduced viability to 30%, while Olaparib pre-treatment restored viability to 68% (38% rescue). Notably, Olaparib alone at 3 µM had minimal effect on cell viability (98% of control), confirming that this concentration is non-toxic. Consistent with the viability rescue, Olaparib pre-treatment also restored NAD+ levels from 35% (combination alone) to 78% of control (43% restoration) and prevented AIF translocation from 78% (combination alone) to 24% nuclear AIF (54% prevention). Similarly, in MDA-MB-231 cells, combination treatment (ED50: 38 µM Etoposide + 40 µM Resveratrol) reduced viability to 25%, and Olaparib pre-treatment restored viability to 65% (40% rescue). Olaparib alone at 3 µM had minimal effect on cell viability (97% of control). Olaparib pre-treatment restored NAD+ levels from 30% (combination alone) to 75% of control (45% restoration) and prevented AIF translocation from 82% (combination alone) to 20% nuclear AIF (62% prevention).

These results provide direct evidence that PARP-1 hyperactivation is essential for the cytotoxic effects of the etoposide–resveratrol combination and establish a causal link between PARP-1 hyperactivation and parthanatos-mediated cell death.

## 4. Discussion

Our study unequivocally demonstrates a potent synergistic antitumor effect of combining etoposide and resveratrol in human breast cancer cell lines, a finding of substantial clinical relevance given the persistent challenges in breast cancer therapy. The observed synergism, consistently evident in both ER-positive (MCF-7) and triple-negative (MDA-MB-231) breast cancer cells, suggests a broad therapeutic applicability, particularly for aggressive and hard-to-treat subtypes like TNBC. This is a critical advancement, as TNBC often lacks targeted therapies and is associated with poor prognosis and high recurrence rates [45,46].

The Chou–Talalay quantitative analysis revealed consistent and robust synergism across multiple effect levels in both cell lines. In MCF-7 cells, CI values ranged from 0.75 (ED50: 45 µM etoposide + 48 µM resveratrol) to 0.62 (ED90: 90 µM etoposide + 96 µM resveratrol), while in MDA-MB-231 cells, CI values ranged from 0.71 (ED50: 38 µM etoposide + 40 µM resveratrol) to 0.58 (ED90: 76 µM etoposide + 80 µM resveratrol). The progressive decrease in CI values from ED50 to ED90 indicates that the synergistic effect strengthens at higher degrees of cell viability inhibition, suggesting that the combination becomes increasingly effective as drug concentrations increase. This dose-dependent enhancement of synergism is particularly notable and suggests that the combination may be especially valuable in achieving high levels of tumor cell killing. The lower CI value in MDA-MB-231 cells (0.58 at ED90) is particularly encouraging, suggesting that this combination might be exceptionally effective against this aggressive and challenging breast cancer subtype. This finding opens new avenues for therapeutic development, especially considering the limited treatment options currently available for TNBC patients.

Etoposide, a cornerstone of many chemotherapy regimens, primarily functions by inducing DNA damage and subsequently triggering apoptosis [47,48]. However, the development of resistance to apoptosis is a common mechanism by which cancer cells evade chemotherapy, leading to treatment failure [49,50]. Our findings reveal that the combination of etoposide with resveratrol not only enhances cytotoxicity but crucially redirects the mode of cell death towards parthanatos [51,52]. The hyperactivation of PARP-1, evidenced by the significant increase in NAD+ depletion, stands as a central event in this process [53,54]. While PARP-1 is vital for DNA repair, its excessive activation becomes cytotoxic by rapidly depleting cellular NAD+ and ATP, leading to an energetic crisis and subsequent cell demise [55,56]. The quantitative decreases in NAD+ levels (approximately 65% reduction in MCF-7 and 70% in MDA-MB-231) with the combination treatment, far exceeding those of single agents, underscore the robust induction of this pathway [57]. Critically, our Olaparib, PARP inhibitor, rescue experiments provide direct evidence that PARP-1 hyperactivation is not merely associated with but is essential for the cytotoxic effects of the etoposide–resveratrol combination. Pre-treatment with Olaparib (3 µM) at ED50 concentrations significantly rescued cells from combination-induced death (38% rescue in MCF-7 and 40% rescue in MDA-MB-231), restored NAD+ levels to near-control values (43–45% restoration), and prevented AIF translocation to the nucleus (54–62% prevention), establishing a causal link between PARP-1 activation and parthanatos-mediated cell death. This represents a significant advance over previous correlative studies and provides strong mechanistic support for targeting PARP-1 in combination therapy approaches.

The concentration of 3 µM used in this study was specifically chosen to ensure PARP inhibition without inducing off-target toxicity, as higher concentrations (≥10 µM) have been reported to cause cell death independent of PARP inhibition [58]. Our findings that Olaparib alone at 3 µM had minimal effect on cell viability (97–98% of control in both cell lines) confirms the non-toxic nature of this concentration while still effectively inhibiting PARP activity. The robust rescue effect observed with Olaparib pre-treatment—restoring cell viability by 38–40%, NAD+ levels by 43–45%, and preventing AIF translocation by 54–62%—provides compelling evidence that PARP hyperactivation is the critical mediator of the etoposide–resveratrol combination’s cytotoxic effects. Collectively, the ability of Olaparib to reverse these effects further supports that the observed cell death occurs predominantly through a PARP-dependent parthanatos pathway. These results therefore provide mechanistic evidence for the involvement of parthanatos in the synergistic antitumor activity of etoposide and resveratrol.

Resveratrol, a natural polyphenol, has garnered significant attention for its multifaceted anticancer properties, including its ability to modulate various signaling pathways involved in cell proliferation, inflammation, and oxidative stress [59,60]. Our data suggest that resveratrol, when combined with etoposide, acts as a potent sensitizer, exacerbating the cellular stress induced by etoposide and pushing cancer cells towards PARP-1 hyperactivation and parthanatos. The precise mechanisms by which resveratrol contributes to this enhanced parthanatic response are likely complex and multi-factorial. Resveratrol is known to influence cellular metabolism, induce oxidative stress, and directly or indirectly modulate DNA repair pathways [61,62]. It is plausible that resveratrol either enhances the DNA-damaging effects of etoposide, thereby increasing the initial trigger for PARP-1 activation, or directly interferes with the cellular machinery that would normally mitigate PARP-1 hyperactivation. Further investigations into the specific upstream signaling cascades and protein–protein interactions that lead to this robust PARP-1 hyperactivation in the presence of both compounds would provide invaluable mechanistic insights [63].

Furthermore, our qRT-PCR analysis provided crucial insights into the transcriptional regulation of parthanatos. The significant upregulation of PARP-1 and AIF mRNA expression in combination-treated cells (3.5–4.2 fold for PARP-1 and 2.8–3.1 fold for AIF) indicates that the synergistic effect extends beyond immediate post-translational modifications, influencing the cellular machinery responsible for initiating and executing parthanatos at the genetic level [64,65]. This dual-level regulation—both at the protein activity level (PARP-1 activation, AIF translocation) and at the gene expression level—underscores the profound and sustained activation of the parthanatos pathway by the combined treatment. This comprehensive induction of parthanatos, involving both enzymatic hyperactivation and increased gene expression of key mediators, likely contributes to the enhanced and sustained cytotoxicity observed [66].

The comprehensive mechanistic investigation further solidifies our understanding of how the etoposide–resveratrol combination exerts its synergistic antitumor effects. This is a pivotal finding, as it strongly differentiates the induced cell death from classical apoptosis and reinforces parthanatos as the predominant mechanism. The observed low or absent levels of cleaved caspase-3/7 in combination-treated cells, despite the potent cytotoxicity, provides compelling evidence for a caspase-independent cell death pathway [67,68]. This is particularly important for clinical translation, as many cancers develop resistance to apoptosis-inducing agents. By activating parthanatos, the etoposide–resveratrol combination offers a novel strategy to bypass apoptosis resistance, making it a potentially effective therapy for a broader range of breast cancer subtypes, including those refractory to conventional chemotherapy.

The mitochondrial function assays provided crucial insights into the cellular energy dynamics during parthanatos. The significant depolarization of mitochondrial membrane potential (MMP) and the drastic depletion of ATP levels (approximately 75% reduction) in combination-treated cells are direct consequences of PARP-1 hyperactivation, which consumes vast amounts of NAD+ essential for ATP production [69,70]. This energetic catastrophe is a hallmark of parthanatos and contributes directly to cell demise [71]. Furthermore, the increased production of reactive oxygen species (ROS) suggests a feedback loop where oxidative stress can further activate PARP-1, exacerbating the parthanatic cascade [72,73]. These findings highlight the central role of mitochondrial dysfunction and oxidative stress in the synergistic action of etoposide and resveratrol.

The DNA damage assays revealed that the combination treatment significantly enhanced DNA fragmentation, as evidenced by increased nucleosome enrichment in the Cell Death Detection ELISA assay. While etoposide is a known DNA-damaging agent, the amplified damage in the presence of resveratrol suggests that resveratrol may either sensitize cells to etoposide-induced DNA damage or that the extensive DNA fragmentation characteristic of parthanatos contributes to the overall DNA damage observed. This amplified DNA damage serves as a potent trigger for PARP-1 hyperactivation, initating the parthanatos pathway [74,75].

The AIF translocation assays, performed using subcellular fractionation and ELISA quantification, provided definitive evidence for the activation of parthanatos. The significant increase in nuclear AIF levels, expressed as the ratio of nuclear to cytoplasmic AIF concentration, in combination-treated cells (78% in MCF-7 and 82% in MDA-MB-231) demonstrates the translocation of AIF from the mitochondria to the nucleus, a hallmark of parthanatos [76,77]. This AIF translocation is directly linked to the extensive DNA fragmentation observed, confirming that the parthanatos pathway is fully activated [78].

Finally, the caspase activity assays provided definitive evidence against the involvement of classical apoptosis as the primary cell death mechanism. The absence of robust caspase-3/7, -8, and -9 activation in combination-treated cells, despite the profound cell death, unequivocally points towards a caspase-independent pathway [79,80]. This is particularly important for clinical translation, as many cancers develop resistance to apoptosis-inducing agents [81]. By activating parthanatos, the etoposide–resveratrol combination offers a novel strategy to bypass apoptosis resistance, making it a potentially effective therapy for a broader range of breast cancer subtypes, including those refractory to conventional chemotherapy.

While our in vitro findings are highly promising, it is imperative to acknowledge certain limitations and outline future research directions. First, the use of established two-dimensional cell lines, while providing a controlled environment for mechanistic studies, may not fully recapitulate the intricate complexity of the tumor microenvironment, including cellular heterogeneity, stromal interactions, and immune responses, which are present in vivo. Additionally, we did not include normal breast epithelial cell lines in this study, which would be important for assessing the selectivity of the observed cytotoxic effects. Therefore, future studies should transition to more physiologically relevant models, such as three-dimensional (3D) cell culture systems (e.g., spheroids, organoids), patient-derived xenografts (PDXs), and in vivo animal models. These models will be crucial for validating our findings, assessing the efficacy and safety of this combination therapy in a more complex biological context, evaluating potential systemic toxicities, and determining the selectivity of the combination for cancer cells versus normal cells.

Moreover, detailed pharmacokinetic and pharmacodynamic studies are essential to determine optimal dosing regimens, administration routes, and potential drug–drug interactions in vivo. Understanding the bioavailability, metabolism, and distribution of both etoposide and resveratrol, especially when co-administered, will be critical for translating these preclinical findings into clinical applications. Future research should also delve deeper into the specific molecular targets and signaling pathways that are synergistically modulated by the etoposide–resveratrol combination, beyond the direct activation of parthanatos. This could include investigating their effects on other cell death pathways, autophagy, or the tumor microenvironment. The potential for this combination to overcome existing drug resistance mechanisms in breast cancer, particularly in TNBC, also warrants extensive investigation.

The inclusion of Olaparib, PARP inhibitor, rescue experiments in this study provides definitive proof of causality, demonstrating that blocking PARP activity significantly attenuates the cytotoxic effects of the etoposide–resveratrol combination. This mechanistic validation strengthens the evidence for parthanatos as the primary mode of cell death and supports the therapeutic potential of targeting PARP-1 hyperactivation in breast cancer treatment.

In conclusion, our study demonstrates that the combination of etoposide and resveratrol exerts a synergistic antitumor effect in human breast cancer cell lines by robustly activating the parthanatos cell death pathway. This novel combination therapy effectively reduces cell viability and proliferation, and induces parthanatos through both protein activation and gene expression modulation. These findings suggest that targeting the parthanatos pathway with etoposide and resveratrol could offer a promising therapeutic strategy for breast cancer, particularly for overcoming resistance to conventional therapies and addressing aggressive subtypes like TNBC. Further preclinical and clinical investigations are essential to translate these encouraging in vitro results into effective treatment options for breast cancer patients.

## 5. Conclusions

This comprehensive study provides compelling evidence for the synergistic antitumor effect of etoposide and resveratrol in human breast cancer cell lines through the activation of the parthanatos cell death pathway. Our findings demonstrate that this combination therapy offers several key advantages: (1) enhanced cytotoxicity compared to single-agent treatments across multiple effect levels (ED50, ED75, ED90), (2) broad applicability across different breast cancer subtypes, including the challenging triple-negative subtype, (3) activation of a caspase-independent cell death mechanism that may overcome apoptosis resistance, and (4) comprehensive disruption of cellular homeostasis through mitochondrial dysfunction, energy depletion, and oxidative stress.

The mechanistic insights gained from this study reveal that the synergistic effect operates at multiple levels, from transcriptional upregulation of key parthanatos mediators (PARP-1: 3.5–4.2 fold; AIF: 2.8–3.1 fold) to robust protein activation and cellular dysfunction. The quantitative demonstration of synergism through combination index analysis (CI = 0.62–0.75 for MCF-7; CI = 0.58–0.71 for MDA-MB-231), coupled with extensive mechanistic validation, provides a strong foundation for further development of this therapeutic approach.

These results have significant implications for breast cancer treatment, particularly for patients with limited therapeutic options or those who have developed resistance to conventional therapies. The ability to induce parthanatos, a distinct form of programmed cell death, represents a novel strategy that could complement existing treatment modalities and potentially improve patient outcomes [82]. Future research directions should focus on in vivo validation, optimization of dosing regimens, investigation of potential biomarkers for patient selection, and exploration of this combination’s efficacy in overcoming drug resistance [83]. The translation of these promising preclinical findings to clinical applications could ultimately lead to improved therapeutic outcomes for breast cancer patients worldwide.

## Figures and Tables

**Table 1:** Comprehensive Chou–Talalay Analysis: Combination Index (CI) Values at Multiple Effect Levels.

Cell Line	Effect Level (Fa)	Etoposide Conc. (µM)	Resveratrol Conc. (µM)	Combination Index (CI)	Interpretation
MCF-7	ED50 (50%)	45	48	0.75	Synergism
	ED75 (75%)	67	72	0.68	Strong Synergism
	ED90 (90%)	90	96	0.62	Strong Synergism
MDA-MB-231	ED50 (50%)	38	40	0.71	Synergism
	ED75 (75%)	57	60	0.65	Strong Synergism
	ED90 (90%)	76	80	0.58	Strong Synergism

Note: CI < 1 indicates synergism, CI = 1 indicates additive effect, CI > 1 indicates antagonism.

## Data Availability

The data supporting the findings of this study are available within the article.

## References

[1] Sung H., Ferlay J., Siegel R.L., Laversanne M., Soerjomataram I., Jemal A., Bray F. (2021). Global Cancer Statistics 2020: GLOBOCAN Estimates of Incidence and Mortality Worldwide for 36 Cancers in 185 Countries. CA Cancer J. Clin..

[2] Waks A.G., Winer E.P. (2019). Breast Cancer Treatment: A Review. JAMA.

[3] Perou C.M., Sørlie T., Eisen M.B., van de Rijn M., Jeffrey S.S., Rees C.A., Pollack J.R., Ross D.T., Johnsen H., Akslen L.A. (2000). Molecular portraits of human breast tumours. Nature.

[4] Pommier Y., Leo E., Zhang H., Marchand C. (2010). DNA topoisomerases and their poisoning by anticancer and antibacterial drugs. Chem. Biol..

[5] Nitiss J.L. (2009). Targeting DNA topoisomerase II in cancer chemotherapy. Nat. Rev. Cancer.

[6] Hande K.R. (1998). Etoposide: Four decades of development of a topoisomerase II inhibitor. Eur. J. Cancer.

[7] Alpsoy A., Yasa S., Gündüz U. (2014). Etoposide resistance in MCF-7 breast cancer cell line is marked by multiple mechanisms. Biomed. Pharmacother..

[8] Koirala M., DiPaola M. (2024). Overcoming Cancer Resistance: Strategies and Modalities for Effective Treatment. Biomedicines.

[9] Fu Y., Chang H., Peng X., Bai Q., Yi L., Zhou Y., Zhu J., Mi M. (2014). Resveratrol inhibits breast cancer stem-like cells and induces autophagy via suppressing Wnt/β-catenin signaling pathway. PLoS ONE.

[10] Baur J.A., Sinclair D.A. (2006). Therapeutic potential of resveratrol: The in vivo evidence. Nat. Rev. Drug Discov..

[11] Shakibaei M., Harikumar K.B., Aggarwal B.B. (2009). Resveratrol addiction: To die or not to die. Mol. Nutr. Food Res..

[12] Chimento A., Sirianni R., Saturnino C., Caruso A., Sinicropi M.S., Pezzi V. (2016). Resveratrol and Its Analogs as Antitumoral Agents for Breast Cancer Treatment. Mini Rev. Med. Chem..

[13] Pervaiz S. (2003). Resveratrol: From grapevines to mammalian biology. FASEB J..

[14] Alkhalaf M. (2007). Resveratrol-induced apoptosis is associated with activation of p53 and inhibition of protein translation in T47D human breast cancer cells. Pharmacology.

[15] Jin X., Wei Y., Liu Y., Lu X., Ding F., Wang J., Yang S. (2019). Resveratrol promotes sensitization to doxorubicin by inhibiting epithelial–mesenchymal transition and modulating SIRT1/β-catenin signaling pathway in breast cancer. Cancer Med..

[16] Behroozaghdam M., Dehghani M., Zabolian A., Kamali D., Javanshir S., Hasani Sadi F., Hashemi M., Tabari T., Rashidi M., Mirzaei S. (2022). Resveratrol in breast cancer treatment: From cellular effects to molecular mechanisms of action. Cell. Mol. Life Sci..

[17] Cipolletti M., Montalesi E., Nuzzo M.T., Fiocchetti M., Ascenzi P., Marino M. (2019). Potentiation of paclitaxel effect by resveratrol in human breast cancer cells by counteracting the 17β-estradiol/estrogen receptor α/neuroglobin pathway. J. Cell. Physiol..

[18] Radeva L., Yoncheva K. (2025). Resveratrol—A Promising Therapeutic Agent with Problematic Properties. Pharmaceutics.

[19] Gianchecchi E., Fierabracci A. (2020). Insights on the effects of resveratrol and some of its derivatives in cancer and autoimmunity: A molecule with a dual activity. Antioxidants.

[20] Wang Z., Wang F., Tang T., Guo C. (2012). The role of PARP1 in the DNA damage response and its application in tumor therapy. Front. Med..

[21] Andrabi S.A., Kim N.S., Yu S.W., Wang H., Koh D.W., Sasaki M., Klaus J.A., Otsuka T., Zhang Z., Koehler R.C. (2006). Poly(ADP-ribose) (PAR) polymer is a death signal. Proc. Natl. Acad. Sci. USA.

[22] Kang M., Park S., Park S.H., Lee H.G., Park J.H. (2022). A Double-Edged Sword: The Two Faces of PARylation. Int. J. Mol. Sci..

[23] Wang Y., Kim N.S., Haince J.F., Kang H.C., David K.K., Andrabi S.A., Poirier G.G., Dawson V.L., Dawson T.M. (2011). Poly(ADP-ribose) (PAR) binding to apoptosis-inducing factor is critical for PAR polymerase-1-dependent cell death (parthanatos). Sci. Signal..

[24] Wang Y., Dawson V.L., Dawson T.M. (2009). Poly(ADP-ribose) signals to mitochondrial AIF: A key event in parthanatos. Exp. Neurol..

[25] Wang Y., Luo W., Wang Y. (2019). PARP-1 and its associated nucleases in DNA damage response. DNA Repair.

[26] Moura R.D., Mattos P.D., Valente P.F., Hoch N.C. (2024). Molecular mechanisms of cell death by parthanatos: More questions than answers. Genet. Mol. Biol..

[27] David K.K., Andrabi S.A., Dawson T.M., Dawson V.L. (2009). Parthanatos, a messenger of death. Front. Biosci..

[28] Fatokun A.A., Dawson V.L., Dawson T.M. (2014). Parthanatos: Mitochondrial-linked mechanisms and therapeutic opportunities. Br. J. Pharmacol..

[29] Liu L., Li J., Ke Y., Zeng X., Gao J., Ba X., Wang R. (2022). The key players of parthanatos: Opportunities for targeting multiple levels in the therapy of parthanatos-based pathogenesis. Cell. Mol. Life Sci..

[30] Zhou Y., Liu L., Tao S., Yao Y., Wang Y., Wei Q., Shao A., Deng Y. (2021). Parthanatos and its associated components: Promising therapeutic targets for cancer. Pharmacol. Res..

[31] Bai P., Cantó C. (2012). The role of PARP-1 and PARP-2 enzymes in metabolic regulation and disease. Cell Metab..

[32] Mazloum-Ardakani M., Barazesh B., Moshtaghioun S.M., Sheikhha M.H. (2019). Designing and optimization of an electrochemical substitute for the MTT cell viability assay. Sci. Rep..

[33] Celada S.I., Li G., Celada L.J., Kanagasabai T., Lu W., Brown L.K., Mark Z.A., Izban M.G., Ballard B.R., Zhou X. (2024). Castration-resistant prostate cancer is resensitized to androgen deprivation by autophagy-dependent apoptosis induced by blocking SKP2. Sci. Signal..

[34] Zhong H., Song R., Pang Q., Liu Y., Yang X., Liu F., Zhang J., Wang X. (2018). Propofol inhibits parthanatos via ROS–ER–calcium–mitochondria signal pathway in vivo and in vitro. Cell Death Dis..

[35] Chen H.Y., Cheng H.L., Lee Y.H., Yuan T.M., Chen S.W., Lin Y.Y., Chueh P.J. (2017). Tumor-associated NADH oxidase (tNOX)-NAD^+^-sirtuin 1 axis contributes to oxaliplatin-induced apoptosis of gastric cancer cells. Oncotarget.

[36] Jang K.H., Do Y.J., Son D., Son E., Choi J.S., Kim E. (2017). AIF-independent parthanatos in the pathogenesis of dry age-related macular degeneration. Cell Death Dis..

[37] Huang C.T., Huang D.Y., Hu C.J., Wu D., Lin W.W. (2014). Energy adaptive response during parthanatos is enhanced by PD98059 and involves mitochondrial function but not autophagy induction. Biochim. Biophys. Acta.

[38] McMillan E.M., Quadrilatero J. (2011). Differential apoptosis-related protein expression, mitochondrial properties, proteolytic enzyme activity, and DNA fragmentation between skeletal muscles. Am. J. Physiol. Regul. Integr. Comp. Physiol..

[39] Dam A.D., Mitchell A.S., Rush J.W., Quadrilatero J. (2012). Elevated skeletal muscle apoptotic signaling following glutathione depletion. Apoptosis.

[40] Toyoda M., Takagi H., Horiguchi N., Kakizaki S., Sato K., Takayama H., Mori M. (2002). A ligand for peroxisome proliferator-activated receptor gamma inhibits cell growth and induces apoptosis in human liver cancer cells. Gut.

[41] Gurtu V., Kain S.R., Zhang G. (1997). Fluorometric and colorimetric detection of caspase activity associated with apoptosis. Anal. Biochem..

[42] Peterson Q.P., Goode D.R., West D.C., Botham R.C., Hergenrother P.J. (2010). Preparation of the caspase-3/7 substrate Ac-DEVD-pNA by solution-phase peptide synthesis. Nat. Protoc..

[43] Chou T.C. (2010). Drug combination studies and their synergy quantification using the Chou–Talalay method. Cancer Res..

[44] Adhireksan Z., Palermo G., Riedel T., Ma Z., Muhammad R., Rothlisberger U., Davey C.A. (2017). Allosteric cross-talk in chromatin can mediate drug–drug synergy. Nat. Commun..

[45] Jie H., Ma W., Huang C. (2025). Diagnosis, Prognosis, and Treatment of Triple-Negative Breast Cancer: A Review. Breast Cancer.

[46] Inampudi P., Yadlapalli D.C., Gullipalli M. (2024). Clinicopathological profiles of and patterns of recurrence in triple-negative breast cancer patients at a cancer care center in Southern India. Cureus.

[47] Alaaeldin R., Abdel-Rahman I.M., Ali F.E.M., Bekhit A.A., Elhamadany E.Y., Zhao Q.L., Cui Z.G., Fathy M. (2022). Dual topoisomerase I/II inhibition-induced apoptosis and necro-apoptosis in cancer cells by a novel ciprofloxacin derivative via RIPK1/RIPK3/MLKL activation. Molecules.

[48] Wei F., Hao P., Zhang X., Hu H., Jiang D., Yin A., Wen L., Zheng L., He J.Z., Mei W. (2018). Etoposide-induced DNA damage affects multiple cellular pathways in addition to DNA damage response. Oncotarget.

[49] Kulbay M., Paimboeuf A., Ozdemir D., Bernier J. (2022). Review of cancer cell resistance mechanisms to apoptosis and actual targeted therapies. J. Cell. Biochem..

[50] Wein L., Loi S. (2017). Mechanisms of resistance of chemotherapy in early-stage triple negative breast cancer (TNBC). Breast.

[51] Huang P., Chen G., Jin W., Mao K., Wan H., He Y. (2022). Molecular mechanisms of parthanatos and its role in diverse diseases. Int. J. Mol. Sci..

[52] Liang S.P., Wang X.Z., Piao M.H., Chen X., Wang Z.C., Li C., Wang Y.B., Lu S., He C., Wang Y.L. (2023). Activated SIRT1 contributes to DPT-induced glioma cell parthanatos by upregulation of NOX2 and NAT10. Acta Pharmacol. Sin..

[53] Ha H.C., Snyder S.H. (1999). Poly(ADP-ribose) polymerase is a mediator of necrotic cell death by ATP depletion. Proc. Natl. Acad. Sci. USA.

[54] Murata M.M., Kong X., Moncada E., Chen Y., Imamura H., Wang P., Berns M.W., Yokomori K., Digman M.A. (2019). NAD^+^ consumption by PARP1 in response to DNA damage triggers metabolic shift critical for damaged cell survival. Mol. Biol. Cell.

[55] Luo T., Yuan Y., Yu Q., Liu G., Long M., Zhang K., Bian J., Gu J., Zou H., Wang Y. (2017). PARP-1 overexpression contributes to cadmium-induced death in rat proximal tubular cells via parthanatos and the MAPK signalling pathway. Sci. Rep..

[56] Song W., Lee E.E., Park S., Kim H., Lee J., Kim Y., Park J., Choi K., Lee H., Kim S. (2025). Type 1 interferon signature and allograft inflammatory factor-1 contribute to refractoriness to TNF inhibition in ankylosing spondylitis. Nat. Commun..

[57] Chowdhry S., Zanca C., Rajkumar U., Koga T., Diao Y., Raviram R., Liu F., Turner K., Yang H., Brunk E. (2019). NAD metabolic dependency in cancer is shaped by gene amplification and enhancer remodelling. Nature.

[58] Ratanaphan A. (2025). Combinative treatment of the PARP inhibitor olaparib and antimetastasis ruthenium(II)-arene compound RAPTA-T for triple-negative BRCA1 wild-type breast cancer cells. Int. J. Mol. Sci..

[59] Ribeiro E., Vale N. (2024). The role of resveratrol in cancer management: From monotherapy to combination regimens. Targets.

[60] Singaravelan N., Tollefsbol T.O. (2025). Polyphenol-based prevention and treatment of cancer through epigenetic and combinatorial mechanisms. Nutrients.

[61] Varoni E.M., Lo Faro A.F., Sharifi-Rad J., Iriti M. (2016). Anticancer molecular mechanisms of resveratrol. Front. Nutr..

[62] Vervandier-Fasseur D., Latruffe N. (2019). The potential use of resveratrol for cancer prevention. Molecules.

[63] Suskiewicz M.J., Munnur D., Strømland Ø., Yang J.C., Easton L.E., Chatrin C., Zhu K., Baretić D., Goffinont S., Schuller M. (2023). Updated protein domain annotation of the PARP protein family sheds new light on biological function. Nucleic Acids Res..

[64] Gupta G., Afzal M., Moglad E., Goyal A., Almalki W.H., Goyal K., Rana M., Ali H., Rekha A., Kazmi I. (2025). Parthanatos and apoptosis: Unraveling their roles in cancer cell death and therapy resistance. EXCLI J..

[65] Hong S.J., Dawson T.M., Dawson V.L. (2004). Nuclear and mitochondrial conversations in cell death: PARP-1 and AIF signaling. Trends Pharmacol. Sci..

[66] Zong L., Liang Z. (2023). Apoptosis-inducing factor: A mitochondrial protein associated with metabolic diseases—A narrative review. Cardiovasc. Diagn. Ther..

[67] Kroemer G., Galluzzi L., Vandenabeele P., Abrams J., Alnemri E.S., Baehrecke E.H., Blagosklonny M.V., El-Deiry W.S., Golstein P., Green D.R. (2009). Classification of cell death: Recommendations of the Nomenclature Committee on Cell Death 2009. Cell Death Differ..

[68] Galluzzi L., Vitale I., Aaronson S.A., Abrams J.M., Adam D., Agostinis P., Alnemri E.S., Altucci L., Amelio I., Andrews D.W. (2018). Molecular mechanisms of cell death: Recommendations of the Nomenclature Committee on Cell Death 2018. Cell Death Differ..

[69] Scaduto R.C., Grotyohann L.W. (1999). Measurement of mitochondrial membrane potential using fluorescent rhodamine derivatives. Biophys. J..

[70] Creed S., McKenzie M. (2019). Measurement of mitochondrial membrane potential with the fluorescent dye tetramethylrhodamine methyl ester (TMRM). Methods Mol. Biol..

[71] Rovini A., Heslop K., Hunt E.G., Morris M.E., Fang D., Gooz M., Gerencser A.A., Maldonado E.N. (2021). Quantitative analysis of mitochondrial membrane potential heterogeneity in unsynchronized and synchronized cancer cells. FASEB J..

[72] Sendtner N., Seitz R., Brandl N., Müller M., Gülow K. (2025). Reactive oxygen species across death pathways: Gatekeepers of apoptosis, ferroptosis, pyroptosis, paraptosis, and beyond. Int. J. Mol. Sci..

[73] Moustakli E., Stavros S., Katopodis P., Skentou C., Potiris A., Panagopoulos P., Domali E., Arkoulis I., Karampitsakos T., Sarafi E. (2025). Oxidative stress and the NLRP3 inflammasome: Focus on female fertility and reproductive health. Cells.

[74] Zhang J.H., Xu M. (2000). DNA fragmentation in apoptosis. Cell Res..

[75] Enari M., Sakahira H., Yokoyama H., Okawa K., Iwamatsu A., Nagata S. (1998). A caspase-activated DNase that degrades DNA during apoptosis, and its inhibitor ICAD. Nature.

[76] Daugas E., Nochy D., Ravagnan L., Loeffler M., Susin S.A., Zamzami N., Kroemer G. (2000). Apoptosis-inducing factor (AIF): A ubiquitous mitochondrial oxidoreductase involved in apoptosis. FEBS Lett..

[77] Vande Walle L., Lamkanfi M., Vandenabeele P. (2008). The mitochondrial serine protease HtrA2/Omi: An overview. Cell Death Differ..

[78] Sevrioukova I.F. (2011). Apoptosis-inducing factor: Structure, function, and redox regulation. Antioxid. Redox Signal..

[79] Tong J., Rufli S., Wong W.W. (2023). Measuring caspase activity using a fluorometric assay or flow cytometry. J. Vis. Exp..

[80] Fu G., Chumanevich A.A., Agniswamy J., Fang B., Harrison R.W., Weber I.T. (2008). Structural basis for executioner caspase recognition of P5 position in substrates. Apoptosis.

[81] Ulakcsai Z., Bagaméry F., Vincze I., Szökő É., Tábi T. (2015). Protective effect of resveratrol against caspase-3 activation in primary mouse fibroblasts. Croat. Med. J..

[82] Shen S., Shao Y., Li C. (2023). Different types of cell death and their shift in shaping disease. Cell Death Discov..

[83] Gottesman M.M. (2002). Mechanisms of cancer drug resistance. Annu. Rev. Med..

